# Array comparative genomic hybridization analysis of *Trichoderma reesei *strains with enhanced cellulase production properties

**DOI:** 10.1186/1471-2164-11-441

**Published:** 2010-07-19

**Authors:** Marika Vitikainen, Mikko Arvas, Tiina Pakula, Merja Oja, Merja Penttilä, Markku Saloheimo

**Affiliations:** 1 VTT Technical Research Centre of Finland, P.O. Box 1000, FI-02044 VTT, Espoo, Finland

## Abstract

**Background:**

*Trichoderma reesei *is the main industrial producer of cellulases and hemicellulases that are used to depolymerize biomass in a variety of biotechnical applications. Many of the production strains currently in use have been generated by classical mutagenesis. In this study we characterized genomic alterations in high-producing mutants of *T. reesei *by high-resolution array comparative genomic hybridization (aCGH). Our aim was to obtain genome-wide information which could be utilized for better understanding of the mechanisms underlying efficient cellulase production, and would enable targeted genetic engineering for improved production of proteins in general.

**Results:**

We carried out an aCGH analysis of four high-producing strains (QM9123, QM9414, NG14 and Rut-C30) using the natural isolate QM6a as a reference. In QM9123 and QM9414 we detected a total of 44 previously undocumented mutation sites including deletions, chromosomal translocation breakpoints and single nucleotide mutations. In NG14 and Rut-C30 we detected 126 mutations of which 17 were new mutations not documented previously. Among these new mutations are the first chromosomal translocation breakpoints identified in NG14 and Rut-C30. We studied the effects of two deletions identified in Rut-C30 (a deletion of 85 kb in the scaffold 15 and a deletion in a gene encoding a transcription factor) on cellulase production by constructing knock-out strains in the QM6a background. Neither the 85 kb deletion nor the deletion of the transcription factor affected cellulase production.

**Conclusions:**

aCGH analysis identified dozens of mutations in each strain analyzed. The resolution was at the level of single nucleotide mutation. High-density aCGH is a powerful tool for genome-wide analysis of organisms with small genomes e.g. fungi, especially in studies where a large set of interesting strains is analyzed.

## Background

The filamentous fungus *Trichoderma reesei *is an important protein production host in biotechnology. It secretes naturally high amounts of cellulase and hemicellulase enzymes capable of degrading plant cell wall carbohydrate polymers. The main industrial applications of *Trichoderma *enzymes are in the pulp and paper, textile and feed industries and in the biofuels sector as well as in food processing. Many high-producing strains in use today were generated by classical mutagenesis. However, little data is available on the genomic alterations that have occurred in these strains resulting in improved cellulase production.

A special and important feature of *T. reesei *is that all strains used in industrial production and research are derived from a single natural isolate, QM6a, which was originally isolated in the Salomon Islands during World War II [1]. To enhance cellulase production, *T. reesei *strains have been subjected to extensive random mutagenesis and selection regimes in many research institutes and companies (for a review see [2]). The first cellulase-enhanced mutant QM9123 was isolated at Natick Laboratories, MA, USA by irradiating conidia of QM6a in a linear particle accelerator; this mutant strain produced twice as much cellulase as its parent on cellulose-containing media [3]. The irradiation mutagenesis was continued with QM9123 and the strain QM9414 producing two to four times more cellulase than QM6a was isolated. Further mutagenesis of QM9414 has then produced several high-producing mutant series in a number of laboratories.

Strains developed at Rutgers University, NJ, USA form a separate line of high-producing mutants. Rut-C30, the best characterized and one of the most widely-used *T. reesei *strains, is a member of this series. Rut-C30 was generated by three mutagenesis steps in conditions aiming at the isolation of catabolite-derepressed mutants [4]. The first step was a UV mutagenesis of QM6a and isolation of M7 strain which was further mutagenized with N-nitrosoguanidine (NTG) leading to the NG14 strain [5]. This strain produced several times more cellulases than the parental strain but still showed considerable catabolite repression. NG14 was further UV-mutagenized and Rut-C30, a catabolite repression deficient mutant, was isolated [4]. Different mutagenesis programs have created several mutant lines in which each mutant has improved ability to produce proteins as compared to its parental strain. The mutant series form a unique pedigree of strains in which a large number of gene alterations affecting protein synthesis and secretion ability are expected to be found. Analysis of mutations in the high-producing *T. reesei *strains would open up understanding of the mechanisms underlying the efficient cellulase production, and would enable targeted genetic engineering for improved production of proteins in general.

Rut-C30 is the best characterized *T. reesei *strain. The strain is known to have a deletion of ~ 2.5 kb in the *cre1 *gene mediating glucose repression [6,7] and a frameshift mutation in the glucosidase II alpha subunit gene [8]. Furthermore, a deletion of 85 kb in NG14 and Rut-C30 in the scaffold 15 has been identified [7]. Recently, genome sequencing of Rut-C30 and its parental strain NG14 was reported [9]. Using massive parallel sequencing 223 single nucleotide mutations, 15 small deletions or insertions and 18 larger deletions were identified in these strains. Additionally, electrophoretic karyotyping and gene mapping have revealed differences in size of chromosomes in Rut-C30 as compared to QM6a and recombination events between chromosomes [10,11]. No mutations have been reported in other strains but electrophoretic karyotyping of several *T. reesei *strains showed that chromosomal rearrangements have occurred in many strains, e.g. the smallest chromosome of QM9414 differed in size from that of QM6a. Furthermore, mapping by hybridization with random clones revealed additional rearrangements between the strains [10].

Comparative genomic hybridization (CGH) was originally developed for detecting and mapping copy number variations (gains and losses) in cancer research [12]. Nowadays, array-format CGH (aCGH) enables much higher resolution in analysis of copy number differences [13]. Various aCGH platforms are available, from bacterial artificial chromosomes (BAC) to cDNA clones and oligonucleotide-based formats [14-16]. Oligonucleotide aCGH has been successfully explored, and several commercial platforms are available ranging up to 2.1 million oligonucleotide probes (currently the highest density microarray platform) on a single slide. However, the large size of mammalian genomes makes it impossible to perform whole genome aCGH at a high resolution in a single experiment. In organisms with smaller genomes, the oligonucleotide tiling arrays allow even a single nucleotide resolution on a single chip.

**Figure 1:**
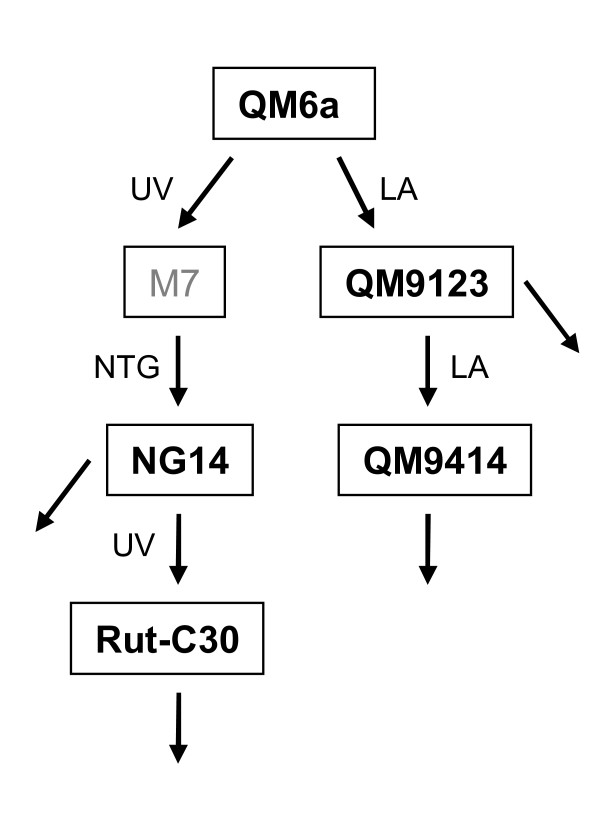
**Genealogy of strains used in this study**. Mutagens used are marked as LA (linear particle accelerator), UV (UV light) and NTG (N-nitrosoguanidine).The gray colour used for strain M7 indicates that M7 was not included in this study. Arrows from strains onwards indicate further mutagenesis of the strains to development of high-producing strains.

In this work we studied the genomic alterations in high cellulase-producing mutants of *T. reesei*. We present the aCGH results of four strains (QM9123, QM9414, NG14 and Rut-C30) with improved protein production in comparison to the QM6a reference strain. The genealogy of these strains is presented in Figure 1. Genomic DNA of the strains was isolated, labelled with different fluorochromes and co-hybridized to high-resolution tiling aCGH of 2.1 million oligonucleotide probes. We detected a large number of genomic alterations in each strain, ranging from single nucleotide mutations to large deletions.

## Results

### Array design and analysis

High-resolution tiling array based on the *T. reesei *QM6a genome sequence [17] was designed by RocheNimbleGen [18]. The design with 2,163,898 probes had essentially complete coverage of all areas where unique probes could be selected. The isothermal overlapping oligonucleotide probes of 45-85 nucleotides (nt) had a mean tiling distance of ~ 15 nt measured from start-to-start. The mean length of probes was 50 nt.

ArrayCGH data was analyzed in R using Bioconductor packages (see Methods). Limma [19] was used for detecting probes with significantly changed intensity and DNAcopy [20] for segmentation to detect genome regions with abnormal copy number. Combined limma testing (p-score cut-off >0.001) and segmentation resulted in 184, 283, 6319 and 5974 probes with significantly deviating signals from QM6a in QM9123, QM9414, NG14 and Rut-C30, respectively. These probes were located in 19, 54, 297 and 156 sites in the genomes of QM9123, QM9414, NG14 and Rut-C30, respectively. A vast majority of the detected changes were losses. The combined results of limma testing and segmentation were visually inspected and mutations appearing as false positives were excluded.

**Figure 2:**
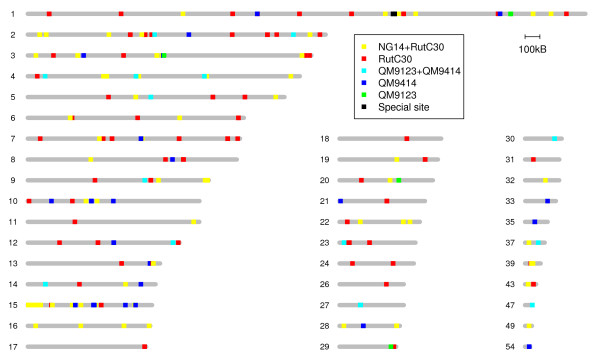
**Location of the mutations identified by aCGH in the *T. reesei *strains**. Scaffolds with mutations are shown and the locations of mutations are marked as squares along the scaffolds (see legend for colour codes referring to the strains). Special site refers to the region of scaffold 1:2460000-2472000.

We present in this study the results of four *T. reesei *strains (Figure 1), but altogether we analyzed 14 strains. However, the majority of these strains are of industrial importance and therefore the results are not published. The industrially relevant strains were derivatives of the four strains discussed here. However, all strains in the data set were included in the interpretation of the CGH data. Having several strains of the same series validated better the strain in which a mutation had originally occurred. This also increased the number of mutations in individual strains, since for some of the probes the detected difference in signal intensity was not statistically significant in a particular strain but showed significant difference in the parent and descendant strain. After manual verification of the analysis results, 20 mutation sites in QM9123, 39 in QM9414, 61 in NG14 and 125 in Rut-C30 were considered as true mutations (Figure 2).

### Resolution of aCGH is at the level of a single nucleotide mutation

The majority of significant change in signal was detected in individual probes or in sets of a few overlapping probes. Therefore, a set of fifty putative mutation sites seen in one or a few probes were randomly chosen for sequencing. This was done to determine whether these sites truly contained a mutation, what types of mutations were detected in the aCGH and what was the false positive rate. The majority of the sites selected for sequencing had losses, but five gains of signal were also included in the data set. Specific oligos designed approximately 300 bp upstream and downstream from the mutation sites were used to amplify a fragment containing a mutation site in the middle of the fragment. Fragments were amplified both from the mutant and from QM6a in order to compare the amplified PCR products. In 37 cases a PCR fragment of correct size was amplified from both strains (34 sites with loss of signal and 3 sites with gain of signal). The fragments both from the mutant and from QM6a were sequenced to rule out mistakes in the published QM6a genome [17]. The majority of the sequenced sites (76%) had single nucleotide substitutions (Tables 1 and 2 and Additional files 1 and 2). Five of the sequenced sites were false positives; two of these sites were from gain signal and three were from loss signal. The false positive rate for all sequenced sites was thus 13.5%. Although two gains were false positives, one such site had a single nucleotide substitution. The false positive rate of sequenced sites with loss signal was 8.8%. In some cases no specific fragments were obtained either from the mutant strain or from QM6a. These sites were specifically located in regions with low CG% and less dense probe design. Sequencing results confirmed that the changes in the probe signals reflected true mutations and that the resolution of the high-density tiling array was at the level of single nucleotide mutation.

**Table 1:** List of mutations in strains QM9123 and QM9414.

Strain	Scaffold	Probe start	Probe end	Gene ID	Element	Gene ID	Element	Position	Mutation
Deletions:									
QM9123	1	2470507	2470997						
QM9414	1	2469100	2470997	103044	promoter				
QM9123 and QM9414	27	140136	141896	5645	IN	68956	IN		
Chromosomal breakpoints:									
QM9123 and QM9414	4	1190121	1190131	76018	IN			1190139	t(14;4)(118472;1190139)
QM9123 and QM9414	9	787736	787744	107460	IN			787779	t(9;27)(787779;140159)
QM9123 and QM9414	14	118442	118456	108962	promoter			118472	t(14;4)(118472;1190139)
QM9123 and QM9414	27	140136	141896	5645	IN	68956	IN	140159	t(9;27)(787779;140159)
Other mutation sites:									
QM9123	1	3252872	3253411	54781	promoter	43191	IN exon		
QM9123	3	918096	918096	104335	IN not spesified				
QM9123	20	399356	399526	35386	IN exon				
QM9123	29	346765	346765	111849	promoter				
QM9123 and QM9414	2	854243	854243	120127	promoter				
QM9123 and QM9414	2	1789354	1789390	2439	promoter			1789397	G→C
QM9123 and QM9414	4	117002	117002	57940	terminator				
QM9123 and QM9414	4	729041	729061						
QM9123 and QM9414	5	828803	828833	58910	promoter			828840	C→A
QM9123 and QM9414	12	978575	978617	108645	promoter				
QM9123 and QM9414	23	31121	31157	81136	IN not specified				
QM9123 and QM9414	30	199387	199481	124022	IN exon			199451	T→A
QM9123 and QM9414	37	89284	89284	70546	promoter				
QM9123 and QM9414	47	49016	49036						
QM9414	1	1546190	1546218	102776	IN not spesified				
QM9414	1	3181743	3181743						
QM9414	2	1085387	1085387						
QM9414	3	376312	376322	104175	IN exon				
QM9414	3	912317	912361	104333	IN exon			912380-912381	TG→CT
QM9414	7	762504	762526	60508	IN intron				
QM9414	8	974648	974656	77661	IN exon			974677-974680	CCGA→ -
QM9414	10	159309	159309	62633	IN intron			159325	T→C
QM9414	10	428575	428613	122036	promoter	78306	promoter	428617	G→ -
QM9414	10	576565	576565	33895	promoter				
QM9414	12	579085	579111	108540	terminator			579119	T→A
QM9414	13	823316	823352					823320	A→T
QM9414	14	762016	762016						
QM9414	15	319892	319892	109278	promoter				
QM9414	15	442981	443029	109313	promoter	65232	promoter	443038-443046	GAGCCACGA → -
QM9414	15	448781	448781	65021	IN exon				
QM9414	15	657953	657953	65141	IN exon				
QM9414	15	726115	726115	79813	IN intron				
QM9414	21	7038	7038	23028	IN exon				
QM9414	28	160600	160622	111645	IN exon				
QM9414	33	152065	152065	52499	promoter				
QM9414	35	66085	66115					66115-66118	AAAT→ -
QM9414	54	27950	27986	124295	promoter			27990-27993	CTTT→-

Strain: the strain(s) in which mutation has been identified, scaffold: scaffold number, probe start and probe end: coordinates of the probe in which the mutation is seen (probe start is the first probe and probe end is the last probe in which the mutation is seen), gene id: the gene according to the protein id in *T. reesei *database v2.0 [17]. Gene id is included if a mutation is considered to be located in one of the gene elements: coding region (IN), promoter or terminator. IN exon or IN intron is marked if the site is sequenced or if the probe coordinates allow reliable location of a mutation site in the exon/intron. If the probe coordinates hit near an exon/intron boundary it is marked as IN not specified. Note that a mutation may affect two genes. Position: the exact coordinate of the mutation if sequenced. Mutation: description of the mutation (t(x;x)(y;y) is a translocation and Δ a deletion).

**Table 2:** List of new mutations identified in strains NG14 and Rut-C30.

Strain	Scaffold	Probe start	Probe end	Gene ID	Element	Position	Mutation	AA change	Annotation
NG14	1	2461556	2470997	103044	IN				Unknown
NG14 and RutC30	1	2408108	2408118	103031	promoter				Unknown
NG14 and RutC30	1	2613321	2613321	53105	promoter				DNA polymerase
NG14 and RutC30	1	3525470	3525482	53811	IN exon	3525497	G→A	Ser_73_→Leu	Clathrin adaptor complex protein
NG14 and RutC30	2	546684	546684			546703	t(4;2)(1204862;546703)		
NG14 and RutC30	3	54311	54311	104067	IN not specified				Unknown
NG14 and RutC30	3	869920	870246						
NG14 and RutC30	4	748146	748270			748277	t(4;22)(748277;139515)		
NG14 and RutC30	4	1204838	1204852	36543	IN	1204862	t(4;2)(1204862;546703)		STE-like zinc finger-containing protein
NG14 and RutC30	10	391916	391922	78301	promoter				Cation efflux protein
NG14 and RutC30	19	385931	385937	80592	IN intron	385954-385957	CCCA→ -		Unknown
NG14 and RutC30	22	139404	139508			139476-139515	t(22;48)(139476;1667), t(4;22)(748277;139515), Δ139477-139514		
NG14 and RutC30	48					1667	t(22;48)(139476;1667)		
RutC-30	2	1614064	1614353	120231	promoter				G protein beta WD40 repeat
RutC-30	7	1019641	1019681	121453	IN exon				Peptidase M, zinc metallopeptidase
RutC-30	12	1014151	1014151						
RutC-30	19	576820	577720	72076	IN	577175-577587	Δ577175-577587		Fungal transcription factor

See Tables 1 and 3 for description. The translocation breakpoint in the scaffold 48 does not have aCGH coordinates since the mutation site was not detected in aCGH but was identified only by LM-PCR as a breakpoint.

### Identification of translocations

When sequencing the putative point mutation sites, amplification of some of the regions resulted in a fragment of correct size in the strain QM6a, but no specific product was obtained from the mutant strain. These mutation sites were considered as putative translocation breakpoints. In order to study this hypothesis we used ligation-mediated PCR (LM-PCR) for genome walking [21]. LM-PCR is a single-sided PCR method that initially requires specification of only one primer binding site, the second being defined by the ligation-based addition of a unique DNA linker.

We identified four translocation breakpoints in QM9123 and QM9414 (Table 1). The translocation t(14;4)(118472;1190139) disrupts the gene 76018 in the scaffold 4 and the promoter of the gene 108962 in the scaffold 14. The breakpoints were seen in the aCGH data as a loss of signal in one or two probes. The second translocation in QM9123 and QM9414 is an unbalanced translocation. The location of the probes with loss of signal at the position 787736-787744 in the scaffold 9 was used as a starting point for LM-PCR. The amplified fragment revealed a translocation (9;27)(787779;140159). The translocation site in the scaffold 27 is just downstream of a deletion of 1.7 kb (see below) and the breakpoint is in the region of the same probes (27:140136-141896) that detect the large deletion. In addition to the two genes disrupted in the scaffold 27 (see next section in results), the translocation disrupts the gene 107460 encoding a GTPase-activating protein in the scaffold 9.

Five translocation breakpoints were identified in Rut-C30 and NG14 (Table 2). The translocation t(4;2)(1204862;546703) disrupts a STE-like transcription factor gene 36543 in the scaffold 4, and there are no additional ORFs (open reading frame) in the vicinity of the translocation site. The other breakpoints, t(22;48)(139476;1667) and t(4;22)(748277;139515) belong to the same translocation event and identify both translocation breakpoints in the scaffold 22. The breakpoint in the scaffold 22 has resulted in t(22;48) and t(4;22) and a deletion of 38 bp in the scaffold 22 (22:139477-139514). These breakpoints are in a region with no ORFs nearby.

### Mutations in strains QM9123 and QM9414

**Figure 3:**
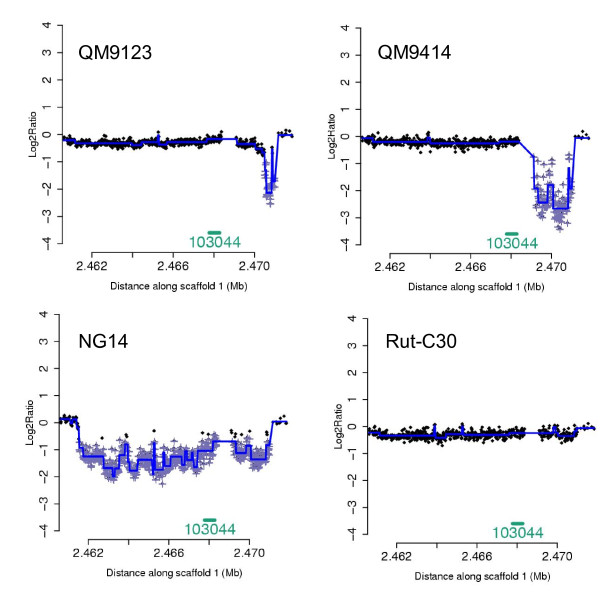
**Region of scaffold 1:2460000-2472000 in four *T. reesei *strains**. The region of scaffold 1:2460000-2472000 of four *T. reesei *mutants. Log2 ratio (y-axis) is plotted against the genome coordinates (x-axis).· = probe in aCGH, + = significant probe and blue line = identified segments.

Twenty mutations were detected in the strain QM9123 (Tables 1 and 3). Four of these were QM9123 specific mutations not found in QM9414 and were thus considered to be independent of the mutagenesis and selection steps. However, we concluded these mutations to be true and included them in the list, since they were also detected in other strains derived directly from QM9123 (data not shown). In the strain QM9414, 23 novel mutations were detected in addition to the 16 mutations originating from QM9123 (Tables 1 and 3). Two large deletions were detected both in QM9123 and QM9414. The 1.7 kb deletion which results from the unbalanced translocation in the scaffold 27 (see above) truncates two genes. The first gene (85645) encodes a putative gamma-glutamyltranspeptidase also having homology to a linkomycin-consensing protein. The second truncated gene (68956) is a FAD-dependent oxidoreductase/D-amino acid oxidase. Another large deletion in QM9123 and QM9414 was found in the scaffold 1, in a region with low GC% and containing a few predicted ORFs with short exons and long introns (Figure 3). The probe density was lower in some parts of this region due to repeat DNA. The deletion is smaller in QM9123 (probes 1:2470507-2470997, ~0.5 kb) than in QM9414 (probes 1:2469100-2470997, ~1.9 kb). Interestingly, a large deletion of ~9.4 kb in this same region was detected in NG14 (probes 1:2461556-2470997) (Figure 3). However, no deletion of this particular region was found in Rut-C30. Although the deletions are of different size and have different starting points in different strains, they appear to end at the same point (probe 1:2470997). We attempted to amplify fragments of this region to identify the exact sites of the deletions but we were unable to obtain specific fragments.

**Table 3:** List of genes that have a mutation in a promoter, coding region or terminator in strains QM9123 and QM9414.

Strain	Gene ID	Element	AA change	Annotation
Deletions:				
QM9123	103044	promoter		Unknown
QM9414	5645	IN		Gamma-glutamyltranspeptidase, plays a key role in the gamma-glutamyl cycle, a pathway for the synthesis and degradation of glutathione and drug and xenobiotic detoxification
QM9123 and QM9414	68956	IN		FAD dependent oxidoreductase, D-amino acid oxidase
Chromosomal breakpoints:				
QM9123 and QM9414	76018	IN		CysK Cysteine synthase
QM9123 and QM9414	107460	IN		GTPase activator protein
QM9123 and QM9414	108962	promoter		Unknown, contains a TLDc domain
QM9123 and QM9414	5645	IN		
QM9123 and QM9414	68956	IN		
Other mutation sites:				
QM9123 and QM9414	120127	promoter		Sequence similarity to GATA type zinc finger, asd4, of *N. crassa *which is involved in ascospore development
QM9123 and QM9414	2439	promoter		ARP2/3 complex protein, candidate arc15, required for the motility and integrity of cortical actin patches
QM9123 and QM9414	57940	terminator		Alternative oxidase, mitochondrial
QM9123 and QM9414	58910	promoter		Unknown
QM9123 and QM9414	108645	promoter		Unknown
QM9123 and QM9414	81136	IN not specified		Hypothetical plasmamembrane proteolipid 3 (induced by high salt or low temp)
QM9123 and QM9414	124022	IN exon	Phe_784_→Tyr	Hypothetical protein
QM9123 and QM9414	70546	promoter		Hypothetical poly(A) polymerase
QM9414	102776	IN not spesified		Unknown
QM9414	104175	IN exon		Unknown, orphan
QM9414	104333	IN exon	Val_402 _→Leu	Ankyrin repeat protein
QM9414	60508	IN intron		UV-endonuclease
QM9414	77661	IN exon	frameshift Pro_125_→	Oxysterol-binding protein
QM9414	62633	IN intron		Unknown
QM9414	122036	promoter		Ribosomal subunit S2/S5
QM9414	78306	promoter		Thiosulphate sulfurtransferase
QM9414	33895	promoter		FKBP peptidylprolyl isomerase
QM9414	108540	terminator		Hypothetical cell wall mannoprotein
QM9414	109278	promoter		Glycoside hydrolase family 24, lysozyme-type
QM9414	109313	promoter		Unknown and 65232
QM9414	65232	promoter		Short-chain dehydrogenase/reductase
QM9414	65021	IN exon		Short-chain dehydrogenase/reductase family
QM9414	65141	IN exon		Cytochrome P450
QM9414	79813	IN intron		Mono-oxygenase, FAD binding
QM9414	23028	IN exon		Hypothetical Ca2+ permeable channel
QM9414	111645	IN exon		Unknown, orphan
QM9414	52499	promoter		Zn-finger protein, C2H2 type
QM9414	124295	promoter		Putative cell wall protein with CFEM domain
QM9123	54781	promoter		Class 1 peptide chain release factor
QM9123	43191	IN exon		Unknown
QM9123	104335	IN not spesified		Unknown
QM9123	35386	IN exon		Hypothetical actin interacting protein 3 (AIP3/BUD6)
QM9123	111849	promoter		xyn4, Glycoside hydrolase family 5

Strain: the strain(s) in which a mutation has been identified, gene id: the gene number according to the protein id in *T. reesei *database v2.0 [17], gene element: coding region (IN), promoter or terminator. IN exon or IN intron is marked if the site is sequenced or if the probe coordinates allow reliable location of a mutation site in the exon/intron. If the probe coordinates hit near an exon/intron boundary it is marked as IN not specified. AA change: marked if the mutation site has been sequenced and a mutation changes the amino acid sequence; Annotation: description of the protein.

The rest of the mutations in QM9123 and QM9414 were seen as a change of signal intensity of only one or a few adjacent probes. Excluding sites identified as translocation breakpoints (see above), there were 14 such mutation sites in QM9123 and 23 more in QM9414 (Tables 1 and 3). Locations of mutations were evaluated according to the gene annotations using the filtered models in *T. reesei *database v2.0 [17]. A 1 kb region upstream from a start codon was considered as a promoter and a 250 bp region downstream from a stop codon as a terminator. Of the mutation sites shared by both strains, two were located in the coding region (genes 81136 and 124022), five in the promoter and one in the terminator of a gene (Table 3). Two QM9123-specific mutations were detected as gains. The gain in 1:3252872-3253411 is >500 bp long and locates in the promoter of the gene 54781 coding a class 1 peptide chain release factor and in the coding region of an unknown gene 43191. The second gain (20:399356-399526) covers a ca. 170 bp region of the gene 35386 encoding an actin-interacting protein. The majority of the QM9414-specific mutations hit a gene element; a promoter (8), a coding region (11) or a terminator (1) (Table 3).

### Mutations in strains NG14 and RutC30

We identified a total of 61 mutations in NG14 and 125 in RutC30, of which 65 mutations were Rut-C30 specific (Table 2 and Additional files 1 and 2). Four additional mutations were discovered as NG14-specific (not shown except for a deletion in the scaffold 1). Since NG14-specific mutations were discovered, it is possible that some other mutations detected in NG14 and further on in Rut-C30 have not occurred in the mutagenesis to obtain NG14 but originate from genetic drift. Of the identified mutations in NG14 and Rut-C30, 109 were discovered earlier [9]. Mutations identified both by our aCGH analysis and sequencing study are listed in Additional files 1 and 2 and the novel mutations detected in this study in Table 2. The large 85 kb deletion in the scaffold 15 (15:1555-86603) in NG14 and Rut-C30 [7] was observed in the aCGH data in probes 15:3431-86866. The number of significant probes in Rut-C30 and NG14 is high compared to QM9123 and QM9414 due to this 85 kb deletion, since the vast majority of probes hit this deletion. The deletion in the *cre1 *gene in Rut-C30 [6] was also detected (probes 2:786836-789542). However, the deletion of a single nucleotide in the glucosidase II alpha subunit gene (121351) [8] was not detected. To verify that our Rut-C30 strain did contain the mutation we sequenced the gene and the reported mutation was detected.

We identified 17 mutations not reported earlier (Table 2). NG14 had a large deletion of 9.4 kb in the scaffold 1 (see above). Five mutation sites in NG14 and Rut-C30 were identified as translocation breakpoints (see above). Seven additional point mutations were identified both in NG14 and Rut-C30. Three of these mutations were in the coding regions of three different genes (a putative clathrin complex protein and two genes of unknown function) and three in the promoter regions of other genes. Rut-C30 had four additional mutations not reported earlier. A deletion was found in the scaffold 19 (probes 19: 577146-577570). The region was sequenced and a deletion of 413 bp (19:577175-577587) was verified. This deletion truncates the gene 72076 encoding a fungal specific transcription factor. The deletion occurs in the second exon and shifts the remaining sequence after deletion out of frame. In the scaffold 2 (probes 2:1614064-1614353) there is a ~280 bp region with loss of signal appearing to be a deletion in the promoter region of the gene 120231 encoding a putative G protein with WD40 repeats. A third Rut-C30-specific mutation affecting a gene element is in the gene 121453 encoding a metallopeptidase. In [9] this ORF has a mutation in 7:1019614 but our data had a change of signal in the probes 7:1019641-1019681, placing the mutation at a different position.

We evaluated the fidelity (precision) and completeness (recall) of aCGH results by comparing our Rut-C30 data with sequenced data [9]. Mutations in adjacent nucleotides in [9] were combined into one because aCGH reports mutations in adjacent positions as a single mutation. Precision as a measure of how well aCGH data matched the sequencing data was defined as the number of mutations that were present in both aCGH and sequenced data divided by the total number of mutations found by the sequencing approach. The precision was 0.866, meaning that the sequencing approach missed about 13% of the mutations discovered by the aCGH approach. Recall as a measure how many sequenced mutations were missed in the aCGH analysis was defined as the number of mutations that were found by both approaches divided by the total number of mutations in aCGH. The recall was 0.480. We also studied whether the position of a mutation in the probes affects the detection of a mutation in aCGH. Position-dependency was studied by plotting positions of mutations in probes against probe signals. Of the mutations detected by aCGH, the largest change of signals was in probes with a mutation in the middle of the probe (Additional file 3). However, rather large number of reported mutations was still missed although there were probes containing the mutation in their middle region.

### An 83 kb deletion corresponding to the large deletion in scaffold 15 in NG14 and Rut-C30 does not increase cellulase production in QM6a

**Figure 4:**
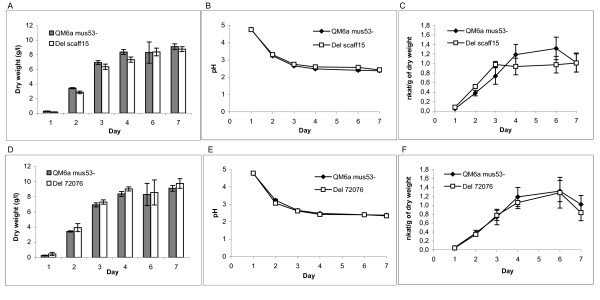
**Growth and cellulase activities of Del scaff15, Del 72076 and their parental strains**. Growth medium TrMM 4% lactose 2% spent grain extract 0.05% peptone pH 4.8. Growth was monitored by measuring the mycelium dry mass (A. Del scaff15 and D. Del 72076) and pH (B. Del scaff15 and E. Del 72076). Cellulase activities of two major cellulases CBHI and EGI was assayed using MUL substrate (C. Del scaff15 and F. Del 72076).

In NG14 and Rut-C30 the deletion of 85 kb in the scaffold 15 abolishes 29 genes from the genome. Since these strains were generated in a mutagenesis and screening procedure to obtain better cellulase producers, we studied whether this deletion is beneficial for cellulase production. The deletion construct was designed according to the aCGH data in which the deletion was detected as having a size of 83 kb instead of 85 kb. The deletion construct to knock out the respective region was constructed by yeast recombinational cloning [22] and transformed in QM6a Δ*mus53 *strain which is the wild-type strain with a deletion of the gene 58509 encoding DNA ligase IV involved in non-homologous end joining of double-strand DNA breaks [23]. This gene has been deleted to increase the frequency of homologous recombination and replacement at the locus to be deleted by the knock-out cassette. The QM6a Δ*mus53 *with the 83 kb deletion in the scaffold 15 (Del scaff15) and the parental strain were cultivated in *Trichoderma *minimal medium (TrMM) [24] supplemented with lactose and spent grain extract (see materials and methods). In this growth medium cellulase genes are induced and cellulases produced [25,26] Growth was monitored by determining biomass dry weight and measuring pH. Growth of Del scaff15 did not differ significantly from the growth of the parental strain (Figure 4). Cellulase activities of two major cellulases, cellobiohydrolase I (CBHI) and endoglucanase I (EGI), were measured as hydrolysis of 4-methylumbelliferyl-β-D-lactoside (MUL) substrate [27] and activities were calculated as nkat/g of dry weight (Figure 4). In the QM6a background the cellulase activities and extracellular protein concentration in general are rather low causing variance between biological replicates. However, if the deletion has any beneficial effect on cellulase production, it is seen as an increase of MUL-activity compared to the QM6a strain. From day 1 to 3 the Del scaff15 appeared to produce slightly more cellulases than the parental strain but from day 4 onwards the activity no longer increased in the deletion strain and the parental strain produced more cellulases on days 4 and 6 (Figure 4). However, the differences were within the range of standard deviation. Cultivations were also carried out in TrMM 2% Solca floc cellulose 0.05% peptone pH 4.8 for 7 days at 28°C. Similar to the results in TrMM lactose spent grain extract medium, there was no significant difference in growth or in cellulase production between Del scaff15 and the parent strain (data not shown).

### Deletion of the gene 72076 does not affect cellulase production in QM6a

Rut-C30 has a deletion in the gene 72076 encoding a fungal transcription factor. Although the N-terminal Cys motif is encoded by the first exon and remains intact in the mutated gene, the deletion and the resulting frameshift most probably leads to a non-functional protein. To study whether deletion of 72076 affected cellulase production, we deleted this gene from the QM6a Δmus53 strain. Construction of the deletion strain was carried out using the same techniques as in constructing the strain Del scaff15 (see above). The deletion strain (Del 72076) and the parental strain were cultivated and dry weight, pH and cellulase production were analyzed as above. Growth of Del 72076 was similar to that of the parental strain, and cellulase production was also similar (Figure 4).

## Discussion

### Overview of the aCGH results

Using a custom made high-resolution tiling aCGH we detected dozens of mutations in all *T. reesei *high-cellulase-producing strains that we analyzed. The aCGH results were validated by sequencing a set of mutation sites and the resolution of our array format was shown to be at the level of a single nucleotide mutation. Altogether, mutations from large deletions to a single nucleotide were detected in all strains analysed. We evaluated how precise and complete the aCGH analysis was by comparing the Rut-C30 data to the sequenced data [9]. Array CGH detected about half of the mutations reported in [9]. On the other hand, we found a number of mutations that were not detected by sequencing [9]. The above suggests that both aCGH and sequencing have their limitations. ArrayCGH may be technically inferior to deep massive parallel sequencing but its lower cost currently justifies its use in species such as *T. reesei*, where there is interest to study many strains of a large phylogeny.

### Various types of mutations were identified in QM9123 and QM9414

We identified over forty mutations of various types in QM9123 and/or QM9414. Considering the number of mutations missed in Rut-C30, the count of mutations in these strains is probably higher than was detected in this study. Nevertheless these mutations are the first to be reported in QM9123 and QM9414. Considering that QM9414 is widely used strain in many laboratories and further mutagenesis of the strain has generated several series of mutants, it is worthwhile to identify mutations in this strain. Sequencing of mutation sites showed that even though several single nucleotide changes were found in QM9123 and QM9414, other types of mutations (e.g. deletion of few nucleotides) were more frequent than in NG14 and Rut-C30. This finding is consistent with the different mutagenesis treatments used to generate the strains [3,4]. Three genes (81136, 124295 and 108540) encoding putative cell wall or plasma membrane proteins contain a mutation in their coding region or in another gene element. Altered cell wall/membrane structure may have been beneficial for spore survival and germination after radiation, or mutations may have been beneficial in the screening procedure itself, being relevant to the improved cellulase production. Two genes involved in cytoskeleton function have a mutation in their coding region; the gene 2439 encodes ARP2/3 complex protein, which is the central regulator of actin cytoskeleton [28] and the gene 35386 encodes actin interacting protein 3 (Aip3/Bud6) which is a regulator of cell and cytoskeleton polarity [29]. These mutations may affect trafficking and secretory pathways in cells. Altogether genes affecting several different functions have been mutated in the QM-strains, and further studies are needed to investigate whether these mutations have effects on protein production.

### Chromosomal translocations were identified in all strains

We identified translocation breakpoints in all strains analyzed. A drawback of aCGH is that, in principle, the method detects only unbalanced chromosomal changes. Balanced chromosomal translocations are seen as a change of signal in a few probes that only identify the site as a mutation site. Although aCGH does not directly identify balanced chromosomal changes, we were able to identify several translocation breakpoints by further analysis of sites with loss of signal using ligation-mediated PCR for genome walking. The presence of breakpoints is consistent with the electrophoretic karyotyping and mapping of genes [10,11], showing that *T. reesei *strains have chromosomal rearrangements. Since QM9123 and QM9414 have been generated by ionizing radiation known to directly induce double strand breakages (DSB), it is not surprising to find translocations in these strains. It is probable that more rearrangements would be found if all the mutation sites detected were analyzed in detail. NG14 and Rut-C30 have undergone other mutagenesis treatments not considered to cause DSB directly, and yet these strains also have translocations. However, all mutagenesis treatments may trigger DNA repair processes and enhance chromosomal recombination, which may lead to rearrangements [30]. Chromosomal rearrangements also occur independently of the mutagenesis agent, and these rearrangements play an important role in the genome evolution. Possibly the lack of a sexual cycle and no need for meiotic pairing of chromosomes may increase the tolerance of genome rearrangements. Palindromic AT-rich regions (PATRRs) have been found to mediate genomic instability, thereby contributing to translocations, deletions and amplifications [31]. One breakpoint identified in this study, t(22;48)(139476;1667) in NG14 and Rut-C30, had occurred in the AT-rich region in one scaffold (scaff 48) and a palindromic sequence was a few base pairs upstream from the breakpoint in another scaffold (scaff 22).

### Scaffold 1 contains a region with deletions in several strains

A specific region in the scaffold 1 appears to be prone to mutations. Deletions of various sizes have occurred in the region in several strains. These deletions are not in accordance with the pedigree in all strains although other mutations in these strains verify that the pedigree is correct. The deletions appear to be due to genetic drift independent from mutagenesis in a region prone to chromosomal rearrangements. In all strains containing a deletion the end point of the deletion is the same probe 1:2470997. This is the last specific probe before a 100 bp region of repeat DNA (CCCTAA)_n _followed by a short gap in the genome sequence. Since repeat sequences act as sites for recombination [30], deletions may have occurred in this repeat region although it is not detected correctly in aCGH. The starting point of deletions varies in the different strains. There is no repeat DNA within a distance of 200 bp from the start point of the deletion in NG14 or QM9123, but in QM9414 the starting point of the deletion is in the region with repeat DNA (TAAAA)_n_.

### New mutations were identified in NG14 and Rut-C30

Using aCGH we identified 126 mutation sites in Rut-C30 and NG14; 109 mutations were the same as reported in [9] and 17 were new ones. The new mutations include 5 translocation breakpoints, three deletions and nine other mutations. We consider these mutations to be true mutations missed in the sequencing since we identified translocations precisely and sequenced three other mutation sites and they all contained a mutation. The new mutations hit some putatively interesting genes: the gene 53811 encoding clathrin adaptor complex protein and the genes 72076, 36543 and 120231 encoding proteins with domains implicated in transcription regulation and signal transduction. As expected, many single nucleotide mutations were missed in aCGH. However, there are also differences in identifying large deletions. Interestingly, the deletion of 9.4 kb in NG14 in the scaffold 1 was missing in the Illumina sequencing data and instead a deletion of 1 kb was identified about 7 kb downstream in the same scaffold in NG14 [9]. Our Rut-C30 strain also contained a deletion not found in the sequencing data in the scaffold 19 and another additional deletion in the scaffold 2. Ten deletions found in the sequencing but missing in aCGH data are located in the regions lacking probes in aCGH due to repeat DNA, which explains why the deletions were missed. However, deletions found in the sequencing in small scaffolds of Rut-C30 are located in regions with normal probe spacing in aCGH. Possibly probe design in these scaffolds is not unique after all and therefore deletions are missed. Known deletions in *cre1 *and in the scaffold 15 in Rut-C30 [6,7] were identified in the aCGH data. The deletion in the scaffold 15 was seen as a size of 83 kb instead of 85 kb because of an AT-rich region in the start of the deletion. Since our array design included only the unique probes there were virtually no probes for the first 3400 bp in the scaffold 15. Therefore the deletion reported to start at 15:1555 was seen in aCGH from 15:3431 onwards.

### Factors affecting aCGH signal

The known point mutation in the glucosidase II alpha subunit gene in Rut-C30 [8] was not detected in aCGH. This observation made us conclude that although aCGH detects single nucleotide mutations the method is unable to identify all of them. This conclusion was then verified by the sequencing data [9]. Microarray hybridization with known sequence mismatches has been used to determine guidelines for array design mostly on short oligonucleotides but also with longer 50-60 mer probes. Altogether, mismatches are more difficult to detect with long probes than with short ones. Strong position-dependent effects have been found; mismatches near the middle of probes reduce the signal more than those near the end of probes [32,33]. Mismatches near the 5' end have a greater effect on hybridization than mismatches the same distance from the 3' end [33]. Mismatches in the 3' end are least effective because probes are attached to the surface by their 3' ends and the close vicinity of microarray surface influences the stringency in hybridization [34]. In principle, in our custom design with a mean length of 50 mer probes and with 15 bp tiling, each nucleotide is in at least three overlapping probes. This tiling should often overcome the problems of position-dependency since a mismatch hits in different positions in different probes. Position-dependency may explain why a mutation was often detected only in one probe (significant change of signal) although the same mutation was also in adjacent probes (no change in signal or the change was below the cut-off level). However, in practice the probe distribution is uneven in many parts of the genome and therefore not all nucleotides are present in several probes in different positions. Our analysis of position-dependency yielded similar results than [32,33], albeit a number of mutations were missed even though there were probes with a mutation in their middle region in aCGH. Non-specific binding to detect mismatches also depends on the substitutions which they incorporate [33,35,36]. However, the results concerning which nucleotide changes cause the greatest effects vary. Despite the difficulties in identifying point mutations with aCGH, we were still able to detect a rather large number of single nucleotide mutations.

### The deletion of 85 kb in scaffold 15 and the deletion in gene 72076 have negligible effects on protein production

Since the deletion in the scaffold 15 is the most prominent mutation identified in Rut-C30 we wanted to study its effect more thoroughly, namely whether the deletion has an effect on hydrolytic enzyme production. To do that we constructed a knock-out strain corresponding to the deletion in the QM6a background. The wild-type strain was chosen to avoid interference with other mutations. The problem with QM6a is that its natural cellulase production level is low compared to that of the high-producing mutants, causing assaying difficulties. According to our results, this 85 kb deletion had no effect on cellulase production in the conditions studied here.

Genomes can be broadly divided into regions where gene content and order is mostly conserved between closely related species (syntenic blocks, SBs) and regions where it is not conserved (non-syntenic blocks, NSBs). In *T. reesei *and *Aspergillus fumigatus *NSBs show enrichment of particular protein families [37,38]. These protein families are typically enriched in Pezizomycotina [39] and are often found in subtelomeres in *Magnaporthe oryzae *[40]. In *A. fumigatus *NSBs tend to be found in or close to subtelomeres and their genes are particularly short [38]. In *A. oryzae *NSBs probably contain many horizontally transferred genes (HGT) [41] of variable, but mostly fungal, origin [42]. Genes in NSBs are induced in starvation-like conditions [43,44] and at least in *A. oryzae *they have significantly lower expression levels than SB genes even when induced [41,43]. NSBs are thus the regions where the majority of mutations, which result in non-lethal phenotypes, occur. The phenotypic effects of NSBs might be restricted by low gene expression levels, which would allow liberal evolutionary experimentation. Of the 29 genes (Additional file 4) found in the 85 kb deletion in the scaffold 15 (Del scaff15), 14 are from protein families typical of *T. reesei *NSBs [37]. Of these, 11 belong to the most enriched protein families in Pezizomycotina [39]. Eleven of the Del scaff15 genes belong to families found in *M. oryzae *subtelomeres, 18 have additional homologues in *T. reesei *[39] and 20 are shorter than the *T. reesei *average of 1,793 bp. In addition, the gene 109199 is probably a result of horizontal gene transfer from Agaricomycotina [39] as its closest homologues are found in these taxons. With the nearby palindromic AT-rich region (PATRR) the Del scaff15 is thus an example of typical fungal evolution that could easily occur without mutagenesis treatment [7]. As such, taking the above data into account as well as the fact that we found no considerable phenotypic effects for it, the Del scaff15 probably has negligible phenotypic effects in protein production conditions.

Our Rut-C30 has a deletion in the gene 72076 encoding a fungal transcription factor. A deletion in a transcription factor gene in a high-cellulase-producing strain already containing a known beneficial deletion in a transcription factor (*cre1*) encouraged us to knock out the 72076 gene and study its effect on cellulase production. However, the Del72076 did not affect cellulase production at least in the conditions studied here. The fact that the 72076 deletion was not identified by [9] indicates that the deletion has occurred specifically in the Rut-C30 isolate used in our laboratory and that the mutation was generated independently of the mutagenesis. Therefore it is not surprising that the Del72076 does not have a beneficial effect on cellulase production. It is also possible that the deletion was missed by [9] and that the deletion occurred in the mutagenesis after all. Moreover, we cannot exclude the possibility that a mutation could have an effect in synergy with some other mutation(s) that were lacking in the QM6a background. Therefore introducing Del scaff15 and Del 72076 to a mutant strain instead of the wild-type strain might evoke a different effect than what is seen in the QM6a background.

## Conclusions

Using array comparative genomic hybridization we detected dozens of mutations in four cellulase high-producing *T. reesei *strains. High-resolution tiling array format allowed detection even at the level of single nucleotide mutation. In strains QM9123 and QM9414 we report the first mutations identified. In NG14 and Rut-C30 we detected new mutations in addition to the mutations that have been reported earlier. The existing sequencing data of Rut-C30 enabled us to evaluate the aCGH method and to compare the results more thoroughly. We also made knock-out constructs to study whether two deletions identified in Rut-C30 are connected to the improved cellulase production and discuss why the deletions appear to be unrelated to the high-producing ability of cellulases. Overall, this study provides insight into the process of developing not just fungal strains, but microbial strains in general for improved industrial production.

## Methods

### Strains, media, culture conditions and gene nomenclature

*Trichoderma reesei *QM6a (ATCC13631, VTT-D-071262T), QM9123 (ATCC 24449, VTT-D-74068), QM9414 (ATCC 26921, VTT-D-74075), NG14 (ATCC 56767, VTT-D-82189) and Rut-C30 (ATCC 56765, VTT-D-86271,) were obtained from VTT Culture Collection. The host strain for the knock-out constructs was QM6a Δmus53. In this strain the gene 58509 has been deleted (unpublished data). Gene id is according to the protein id in *T. reesei *database v2.0 [17]. For DNA isolation the strains were cultivated in shake flasks in *Trichoderma *minimal medium (TrMM) [24] supplemented with 2% glucose, 0.2% peptone pH 4.8 for 2 days at 28°C. Mycelia were collected by filtering, washed with deionised water and lyophilized. To study cellulase production, the deletion strains and the QM6a Δmus53 strain were cultivated in shake flasks in TrMM supplemented with 4% lactose, 2% spent grain extract 0.05% peptone pH 4.8 for 7 days at 28°C. Culture samples were filtered, mycelia were used to determine the dry weight and culture media was used for enzyme assays and measuring pH. For cellulase production studies strains were also cultivated in TrMM 2% Solca floc 0.05% peptone pH 4.8 for 7 days at 28°C. Culture samples were withdrawn, samples were filtered and culture media was used for assays and measuring pH.

### Isolation of chromosomal DNA

DNA for aCGH was isolated from three replicate cultivations of each strain. DNA was isolated according to [45] with modifications. Lyophilized mycelia (100 mg) were first ground in liquid nitrogen. The mycelium powder was suspended in a buffer containing 200 mM Tris-HCl pH 8.5, 250 mM NaCl, 25 mM EDTA pH 8.0 and 0.5% SDS. RNAse was added to a final concentration of 2.6 mg/ml and samples were incubated at 37°C for at least 30 min. Samples were extracted with phenol-chloroform-isoamyl alcohol (24:1) three times followed by extraction with chloroform-isoamyl alcohol (24:1) three times. Isopropanol was added to a volume of 0.54 to precipitate DNA. DNA pellets were washed once with 70% ethanol, air dried 5 min and suspended in deionised water. Quality and purity of the DNA were analyzed according to the Roche NimbleGen guidelines [18]. DNA concentration was measured with NanoDrop ND-1000 (NanoDrop Technologies Inc. Wilmington, DE, USA). DNA for aCGH had to fulfil the criteria of 260/280 ratio ≥ 1.8 and 260/20 ratio ≥1.9. Integrity of DNA and the absence of RNA contamination were analyzed in 1% agarose gel in which chromosomal DNA migrated as a single band of size >50 kb.

### Array design and aCGH

Design and manufacturing of custom aCGH of 2,163,898 probes (HD2 format) was carried out by Roche NimbleGen (WI, USA) [18]. The aCGH design was based on the *T. reesei *QM6a genome sequence [17,37]. The nomenclature of probes is as follows: scaffold number followed by 5' start position of the probe according to the plus strand (first probe in the design 1:12 and the last probe 87:1968). DNA labelling, hybridization and signal quantification were carried out by Roche NimbleGen. Hybridization was carried out as a two-colour reference design (test sample labelled with Cy3 and reference with Cy5) with three repeats for each strain and using a randomly chosen, non-replacement, QM6a sample as a reference. QM6a was treated identically to other strains i.e. hybridised against QM6a three times. Spatial effects were detected in the data and Roche NimbleGen carried out a 2D lowess spatial normalisation [46]. Further data analysis was carried out by the authors. Data from Roche NimbleGen was analysed with R [47] using Bioconductor [48]. Data was parsed to R with the package Ringo [49] and snapCGH [50]. As the use of variance stabilizing normalisation (VSN) [51] alone was unable to completely remove colour biases from the data (data not shown), the data was first normalised within arrays with lowess using the package Limma (Linear models for microarrays) [19]. VSN was then applied to stabilize low intensity probes. Probes with significantly different signals between the strains were detected with Limma [19]. In brief, an anova-like model was fitted for each probe using the QM6a self-hybridization as intercept and strain differences were detected by fitting contrasts between the strains. A cut-off of p > 0.001 was used. In addition, data averaged over the three repeats of each strain and the QM6a self hybridization subtracted, was segmented with DNAcopy [20] in order to identify groups of probes of interest along the scaffolds. Data analysis of the strains discussed in this article was carried out as a part of larger data set (data not shown). The locations of mutations were evaluated according to the gene annotations using the filtered models in *T. reesei *database v2.0 [17]. A 1 kb region upstream from a start codon was considered as a promoter and a 250 bp region downstream from a stop codon as a terminator. Microarray data presented in this study are available in the GEO database [52] under accession number GSE19606.

### Verification of the mutations

Specific primers (Tm 60°C ± 2°C) were designed using Primer3 v4.0 software [53] to anneal approximately 300 bp upstream and downstream from the mutation site. Fragments of approximately 600 bp were amplified in PCR reactions. DNA templates were the same preparations as used in aCGH. Each fragment was amplified both from the mutant strain and from strain QM6a. Fragments were analyzed in 1% agarose gel, isolated with Qiaquick Gel Extraction Kit (Qiagen N.V. Venlo, The Netherlands) and sequenced in both the forward and reverse direction using the same specific oligos as in the PCR amplification. Sequencing was performed using Big Dye^®^Terminator v3.1 Cycle Sequencing Kit (AB Applied Biosystems, Life Technologies Corporation, CA, USA) and analyzed with 3100 Genetic Analyer (AB Applied Biosystems, Life Technologies Corporation, CA, USA).

Ligation-mediated PCR amplification is based on [21]. Chromosomal DNA was digested with EcoRV, DraI or PvuII and the digestion was purified with Qiaquick PCR Purification Kit (Qiagen N.V. Venlo, The Netherlands). Purified DNA was ligated for 3 hours at RT with PCR linker mix in the reaction containing 10 μM of PCRlinkers I and II with T4 ligase (NEB) (PCRlinker I 5' GCGGTGACCCGGGAGATCTGAATTC 3' and PCRlinker II 5' GAATTCAGATCT 3'). The ligation reaction was purified with QIAquick PCR Purification Kit (Qiagen N.V. Venlo, The Netherlands) and used as template in the first PCR reaction performed with 50 pmol of a specific oligo and 5 pmol of PCRlinker I. The PCR product was then used as a template in the second PCR reaction performed with 25 pmol specific nested oligo and 2.5 pmol PCRlinkerI. Product from the second PCR reaction was analyzed in 1% agarose gel and clear bands were isolated with Qiaquick Gel Extraction Kit (Qiagen N.V. Venlo, The Netherlands) and sequenced with the specific oligos used in the first PCR reaction.

### Construction of *T. reesei *deletion strains

The deletion cassette to delete 83 kb of the scaffold 15 was constructed in the pRS426 plasmid [54] and contained the hygromycin resistance cassette flanked by 1 kb fragments from both sides of the intended deletion. The flanking region fragments were amplified by PCR with the oligos: (5' flanking region forward oligo 5' GTAACGCCAGGGTTTTCCCAGTCACGACGACTAGTCGGCCCAACGCATATTATAG 3' and reverse oligo 5' ATCCACTTAACGTTACTGAAATCTCCAACCTTTTTCCTTCCCCCTTCAG 3'), and (3' flanking region forward oligo 5' CTCCTTCAATATCATCTTCTGTCTCCGACACGGCGAATCTACCACAGTC 3' and reverse oligo 5' GCGGATAACAATTTCACACAGGAAACAGCACTAGTAACCAATTCCCACGCATACT 3-). The deletion cassette was constructed by yeast recombinational cloning [22]. The deletion cassette to knock out the gene encoding 72076 was constructed as above. The oligos for flanking region fragments were: (5' flaking fragment forward oligo 5' GTAACGCCAGGGTTTTCCCAGTCACGACGGACGTCCACAGTGTATCACACAACGAC 3' and reverse oligo 5' ATCCACTTAACGTTACTGAAATCTCCAACGAGGAGAAGTGCTCTCAACC 3'), and (3' flanking fragment forward oligo 5' CTCCTTCAATATCATCTTCTGTCTCCGACGATAGCACTGACGCTAAAGC 3' and reverse oligo 5' GCGGATAACAATTTCACACAGGAAACAGCGACGTCCTTTGGTCCTACAGGATCAAG 3'). The constructions were transformed to QM6a Δmus53 by polyethylene glycol-mediated transformation essentially as described by [24], and transformants were selected for hygromycin resistance on plates containing 125 μg ml^-1 ^of hygromycin B (Calbiochem, EMB Biosciences Inc., La Jolla, CA). The transformants were streaked on the selective medium for two successive rounds and tested by PCR. PCR positives were further verified by Southern blotting in order to show that the transformants contained only one copy the hygromycin gene and that this copy had integrated to the correct site in the genome.

### Analysis of proteins in the culture media

Total protein and activity assays were performed from the culture supernatants after removing the mycelia by centrifugation. Soluble protein from the cultivations was assayed by the Bio-Rad protein assay (Bio-Rad Laboratories, Hercules, CA, USA) using bovine serum albumin (Sigma-Aldrich Chemie GmbH, Steinheim, Germany) as the standard. For analysis of cellulase production, the combined action of the two main cellulase components, cellobiohydrolase I (CBHI) and endoglucanase I (EGI), was assayed based on hydrolysis of the low-molecular-weight substrate 4-methylumbelliferyl-β-D-lactoside (MUL) (Sigma-Aldrich) and by detection of the fluorescent hydrolysis product methylumbelliferone [27].

## Authors' contributions

MV designed and carried out the studies (DNA isolation for aCGH, verification of mutations, constructing deletion strains, cultivation and analysis of constructed strains) and data analysis, participated in the aCGH analysis, and drafted the manuscript. MA developed and carried out the aCGH analysis, participated in other data analysis and drafted the manuscript. TP participated in the design of the study, data analysis and drafting of the manuscript. MO performed statistical analysis of aCGH data and prepared a figure for the manuscript. MP and MS conceived of the study, participated in its design and coordination and helped to draft the manuscript. All authors read and approved the final manuscript.

## Supplementary Material

Additional file 1 **Mutations identified both in aCGH analysis and sequencing **[9]**in NG14 and Rut-C30**. Strain: the strain(s) in which a mutation has been identified, scaffold: scaffold number, probe start and probe end: coordinates of the probe in which the mutation is seen (probe start is the first probe and probe end is the last probe in which the mutation is seen), gene id: the gene according to the protein id in *T. reesei *database v2.0 [17]. Gene id is included if a mutation is considered to be located in one of the gene elements: coding region (IN), promoter or terminator. IN exon or IN intron is marked if the site is sequenced. Note that a mutation may affect two genes. Position: the exact coordinate of the mutation if sequenced. Mutation: description of the mutation. Click here for file

Additional file 2 **Mutations identified both in aCGH analysis and sequencing **[9]**in Rut-C30**. see Additional file 1. Click here for file

Additional file 3 **Position of mutations in probes versus log2 signal**. A. Substitutions not detected by aCGH but reported in [9]. B. Substitutions detected both in aCGH and [9]. C. Deletions not detected by aCGH but reported in [9]. D. Deletions detected both in aCGH and [9]. The average probe length of 50 nt was used as the length of probe. Click here for file

Additional file 4 **Genes of the 85 kb deletion in the scaffold 15 and classification of genes to categories**. Gene identifier number for genome version 2.0 and 1.2, strand, start and end coordinates on scaffold 15, description from [7], whether the [39] protein clustering data set contains a close *T. reesei *homologue for the gene, whether [37] supplementary table 2c specifies the InterPro identifier as representative for NSBs, whether [39] additional file 2 specifies the InterPro domain as Pezizomycotina enriched, whether [40] supplementary table 1 of subtelomeric genes has the corresponding InterPro identifier, whether [38] supplementary table 11 of overrepresented domains in lineage specific regions has the corresponding InterPro identifier, count of ESTs according to [37] and InterPro domain identifiers found in the protein. As [37] supplementary table 2c has some Interpro identifiers both as NSB and SB representative, the count of identifiers was normalised with the count of genes in NSB and SB regions and only those relatively more abundant in NSBs were considered as NSB representatives. In [39] additional file 2 the Interpro identifiers with a negative value in PC1 were counted as Pezizomycotina enriched. According to [39] dataset gene 109199 has homologues only in Agaricomycotina, *Coprinopsis cinerea *CC1G_02170 (Uniprot A8NKF5) being the closest. Click here for file
