# Supplementary material for: Array comparative genomic hybridization analysis of Trichoderma reesei strains with enhanced cellulase production properties
Source: BMC Genomics. 2010 Jul 19;11:441. doi: 10.1186/1471-2164-11-441 (PMC3091638; doi:10.1186/1471-2164-11-441)
Supplement: Additional file 1 — Mutations identified both in aCGH analysis and sequencing [9]in NG14 and Rut-C30. Strain: the strain(s) in which a mutation has been identified, scaffold: scaffold number, probe start and probe end: coordinates of the probe in which the mutation is seen (probe start is the first probe and probe end is the last probe in which the mutation is seen), gene id: the gene according to the protein id in T. reesei database v2.0 [17]. Gene id is included if a mutation is considered to be located in one of the gene elements: coding region (IN), promoter or terminator. IN exon or IN intron is marked if the site is sequenced. Note that a mutation may affect two genes. Position: the exact coordinate of the mutation if sequenced. Mutation: description of the mutation. [file 1471-2164-11-441-S1.PDF]

Strain	Scaffold	Probe start	Probe end	Gene ID	Element	Gene ID	Element	Position	Mutation
NG14 and RutC30	1	1045616	1045616	53492	IN				
NG14 and RutC30	1	2497381	2497381						
NG14 and RutC30	1	3404318	3404366						
NG14 and RutC30	2	80065	80101	74570	IN				
NG14 and RutC30	2	124661	124699	55358					
NG14 and RutC30	2	767672	767690	74765	promoter	103653	promoter		
NG14 and RutC30	2	1901184	1901208	75072	promoter	75074	terminator	1901218	C→T
NG14 and RutC30	3	336578	336782	104161	promoter				
NG14 and RutC30	3	906391	906425	45456	IN intron			906436	C→T
NG14 and RutC30	3	1846630	1846822	104599	terminator	56203	promoter		
NG14 and RutC30	4	509861	509861						
NG14 and RutC30	4	531580	531588						
NG14 and RutC30	4	1771895	1771895	3027	IN				
NG14 and RutC30	5	727328	727367						
NG14 and RutC30	5	1670536	1670536	58561	promoter	46794	terminator		
NG14 and RutC30	6	285806	285842	105874	IN intron			285837, 285840	A→T, G→A
NG14 and RutC30	6	1023451	1023481						
NG14 and RutC30	7	482501	482507	3501	promoter			482522	G→A
NG14 and RutC30	7	772023	772023	60243	IN				
NG14 and RutC30	8	424120	424120	77513	IN exon			424139	C → T
NG14 and RutC30	9	880180	880240						
NG14 and RutC30	9	1194725	1194753	107601	promoter	61874	terminator		
NG14 and RutC30	9	1212221	1212243						
NG14 and RutC30	10	466397	466397	78320	IN				
NG14 and RutC30	11	1117611	1117641						
NG14 and RutC30	13	845343	845363	79405	promoter				
NG14 and RutC30	14	658718	658718	64866	promoter				
NG14 and RutC30	15	3431	86866					85 Kb deletion	
NG14 and RutC30	15	165416	165422	65104	IN exon			165438	T → A
NG14 and RutC30	15	298818	298836						
NG14 and RutC30	15	358009	358009	109285					
NG14 and RutC30	15	458185	458193	109320	promoter	109321	terminator		
NG14 and RutC30	15	721390	721442	64882	terminator				
NG14 and RutC30	16	53650	53676	109432	promoter				
NG14 and RutC30	16	357362	357386	22841	IN				

Strain	Scaffold	Probe start	Probe end	Gene ID	Element	Gene ID	Element	Position	Mutation
NG14 and RutC30	16	648529	648557	40758					
NG14 and RutC30	16	817450	817472	4921	promoter				
NG14 and RutC30	20	340451	340457	67030	promoter				
NG14 and RutC30	22	430660	430682						
NG14 and RutC30	22	475285	475321	67658	promoter	5363	terminator		
NG14 and RutC30	28	29741	29741	69437	IN exon			29750	C→T
NG14 and RutC30	28	384505	384711	69181	promoter			384711	C→T
NG14 and RutC30	32	137350	137366	70071	IN exon			137382	T→C
NG14 and RutC30	37	26179	26179	124172	terminator				
NG14 and RutC30	39	43541	43547	6014	promoter	6015	promoter		
NG14 and RutC30	39	53606	53612						
NG14 and RutC30	43	20830	20842	28409	IN				
NG14 and RutC30	49	27427	27439						
