# Supplementary material for: Array comparative genomic hybridization analysis of Trichoderma reesei strains with enhanced cellulase production properties
Source: BMC Genomics. 2010 Jul 19;11:441. doi: 10.1186/1471-2164-11-441 (PMC3091638; doi:10.1186/1471-2164-11-441)
Supplement: Additional file 2 — Mutations identified both in aCGH analysis and sequencing [9]in Rut-C30. see Additional file 1. [file 1471-2164-11-441-S2.PDF]

Strain	Scaffold	Probe start	Probe end	Gene ID	Element	Gene ID	Element	Position	Mutation
RutC-30	1	143260	143540						
RutC-30	1	628586	628600	1751	promoter				
RutC-30	1	1379801	1379815	73912	terminator				
RutC-30	1	1546900	1546930	102776	promoter			1546930-1546931	CC→TT
RutC-30	1	1830325	1830351	119768	IN				
RutC-30	1	2181431	2181461	54511	promoter				
RutC-30	1	2532116	2532140	103061	IN				
RutC-30	1	3168279	3168289						
RutC-30	1	3628846	3628846	54157	IN				
RutC-30	2	690287	690333	56077	promoter				
RutC-30	2	786836	789542	120117	IN				
RutC-30	2	835113	835141	55887	IN			835149	C→T
RutC-30	2	1377476	1377482						
RutC-30	2	1677174	1677174						
RutC-30	2	1967944	1967944	75105	IN exon			1967974	C→T
RutC-30	3	172983	172983	26255	IN				
RutC-30	3	645572	645596	2583	promoter				
RutC-30	3	1866332	1866364	56726	promoter			1866373	G→C
RutC-30	3	1898162	1898182						
RutC-30	4	63620	63620	58073	IN				
RutC-30	5	543842	543842	105391	IN				
RutC-30	5	1246646	1246668	76515	IN			1246681	C→T
RutC-30	5	1462596	1462612	58790	IN			1462623	C→T
RutC-30	6	298658	298658	59388	IN				
RutC-30	6	739997	739997	106009	IN				
RutC-30	6	1419021	1419053	59146	terminator				
RutC-30	7	83438	83438						
RutC-30	7	508358	508358						
RutC-30	7	567452	567474						
RutC-30	7	1346081	1346441	60458	IN				
RutC-30	7	1413936	1413936	59801	IN				
RutC-30	8	927542	927542						
RutC-30	8	1047469	1047497	107078	IN				
RutC-30	8	1049852	1049852						
RutC-30	9	452476	452476	22294	IN				

Strain	Scaffold	Probe start	Probe end	Gene ID	Element	Gene ID	Element	Position	Mutation
RutC-30	9	828341	828371						
RutC-30	10	8441	8441	78158	IN				
RutC-30	10	300175	300175	78268	promoter				
RutC-30	11	317059	317059	108133	promoter	31118	promoter		
RutC-30	12	213801	213801	63702	IN			213823	C→T
RutC-30	12	472181	472217	79014	IN exon			472221	G→A
RutC-30	13	632123	632131	63935	IN				
RutC-30	13	840655	840655						
RutC-30	14	347915	347915	122689	IN				
RutC-30	15	158898	158912						
RutC-30	15	494823	494837	65106	IN				
RutC-30	17	787313	787341	65773	IN				
RutC-30	18	453354	453396	28731	promoter				
RutC-30	20	147461	147475	22994	IN				
RutC-30	21	319076	319144						
RutC-30	22	52345	52359						
RutC-30	23	50198	50198	110882	IN				
RutC-30	23	66520	66574	81149	promoter			66573	G→A
RutC-30	23	201920	201956	5403	IN				
RutC-30	24	87310	87310						
RutC-30	24	360760	360772	111109	promoter	111110	promoter		
RutC-30	26	315789	315789	123786	IN				
RutC-30	29	366649	366649						
RutC-30	31	56297	56297						
RutC-30	39	38560	38560	82499	IN				
RutC-30	43	56617	56665						
