# Supplementary figures and images for: Array comparative genomic hybridization analysis of Trichoderma reesei strains with enhanced cellulase production properties
Source: BMC Genomics. 2010 Jul 19;11:441. doi: 10.1186/1471-2164-11-441 (PMC3091638; doi:10.1186/1471-2164-11-441)

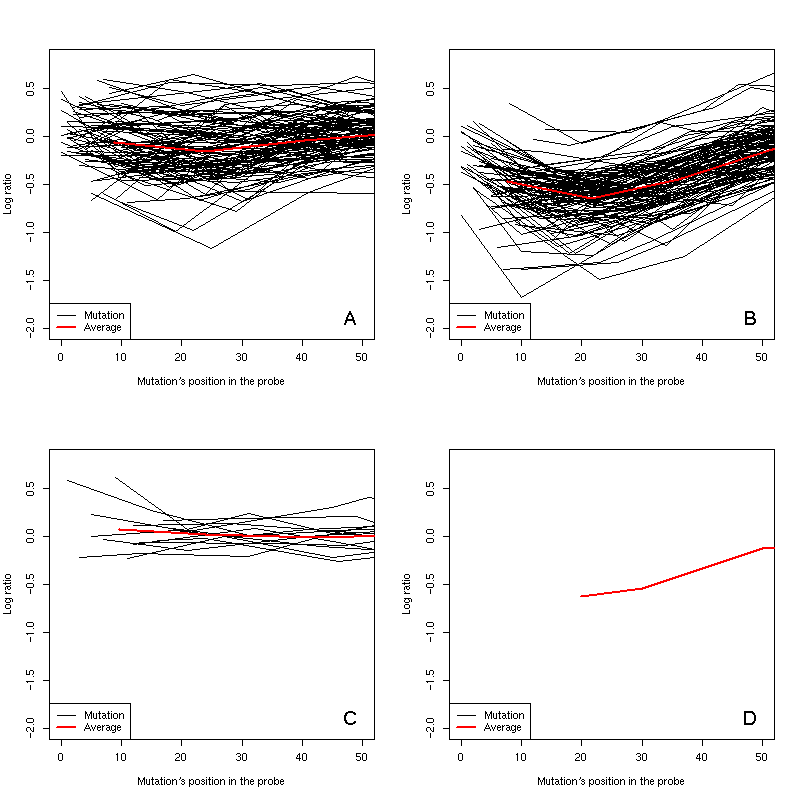

Supplement: Additional file 3 — Position of mutations in probes versus log2 signal. A. Substitutions not detected by aCGH but reported in [9]. B. Substitutions detected both in aCGH and [9]. C. Deletions not detected by aCGH but reported in [9]. D. Deletions detected both in aCGH and [9]. The average probe length of 50 nt was used as the length of probe. [file 1471-2164-11-441-S3.PNG]
