# Supplementary material for: Array comparative genomic hybridization analysis of Trichoderma reesei strains with enhanced cellulase production properties
Source: BMC Genomics. 2010 Jul 19;11:441. doi: 10.1186/1471-2164-11-441 (PMC3091638; doi:10.1186/1471-2164-11-441)
Supplement: Additional file 4 — Genes of the 85 kb deletion in the scaffold 15 and classification of genes to categories. Gene identifier number for genome version 2.0 and 1.2, strand, start and end coordinates on scaffold 15, description from [7], whether the [39] protein clustering data set contains a close T. reesei homologue for the gene, whether [37] supplementary table 2c specifies the InterPro identifier as representative for NSBs, whether [39] additional file 2 specifies the InterPro domain as Pezizomycotina enriched, whether [40] supplementary table 1 of subtelomeric genes has the corresponding InterPro identifier, whether [38] supplementary table 11 of overrepresented domains in lineage specific regions has the corresponding InterPro identifier, count of ESTs according to [37] and InterPro domain identifiers found in the protein. As [37] supplementary table 2c has some Interpro identifiers both as NSB and SB representative, the count of identifiers was normalised with the count of genes in NSB and SB regions and only those relatively more abundant in NSBs were considered as NSB representatives. In [39] additional file 2 the Interpro identifiers with a negative value in PC1 were counted as Pezizomycotina enriched. According to [39] dataset gene 109199 has homologues only in Agaricomycotina, Coprinopsis cinerea CC1G_02170 (Uniprot A8NKF5) being the closest. [file 1471-2164-11-441-S4.PDF]

| JGI v2.0 | JGI v1.2 | Strand | Start | End | Length | Seidl08 description | Arvas07 close homologue | Martinez08 NSB | Arvas07 Pezizomycotina enriched | Rehmeyer06 subtelomeric | Fedorova08 lineage-specific | Count of ESTs | InterPro identifiers |
|---|---|---|---|---|---|---|---|---|---|---|---|---|---|
| 109199 | 36637 | + | 247 | 3990 | 3743 | Rhodanese-like | | | | | | 0 | IPR001763 |
| 43418 | 45000 | - | 4265 | 4450 | 185 | Hypothetical protein | | | | | | 14 | |
| 109201 | 36635 | + | 7135 | 9461 | 2326 | FAD-linked oxidase | x | | | x | x | 0 | IPR006094 |
| 64959 | 30217 | - | 15618 | 16994 | 1376 | Peptidase S26, signal peptidase | x | | | | | 11 | IPR000223,IPR006353,IPR006357 |
| 122778 | 42690 | + | 18217 | 19356 | 1139 | Glycerol dehydrogenase GLD2 | x | x | | x | | 5 | IPR001395 |
| 71817 | 10296 | + | 20262 | 21664 | 1402 | Multidrug resistance protein | x | | | | | 0 | |
| 65215 | 10233 | - | 21775 | 22841 | 1066 | Carbohydrate esterase (family 4), imidase | | | | | | 0 | IPR002509 |
| 65191 | 10310 | + | 25282 | 27143 | 1861 | Maltose permease | x | x | x | x | x | 0 | IPR007114,IPR005829,IPR003663,IPR005828 |
| 109206 | 10183 | - | 27655 | 29638 | 1983 | Heterokaryon incompatibility protein het-6 | x | x | x | x | | 0 | IPR010730 |
| 64906 | 36628 | + | 32246 | 33596 | 1350 | Glucan endo-1,6-β-glucosidase (GH5) | x | | | | | 0 | IPR001547 |
| 49946 | 44997 | + | 35024 | 36114 | 1090 | Glutathione S-transferase | x | | | | | 4 | IPR012335,IPR004045,IPR004046 |
| 65117 | 10169 | + | 36973 | 37308 | 335 | Ankyrin repeat protein | | x | x | | | 0 | IPR002110 |
| 4726 | 36625 | + | 39475 | 41248 | 1773 | Protein of the cytochrome P450 CYP2 family (phenylacetate-2 hydroxylase) | | x | x | x | | 0 | IPR001128 |
| 109211 | 36624 | - | 41375 | 43162 | 1787 | Monocarboxylate transporter | x | x | x | x | x | 0 | IPR007114,IPR011701 |
| 122780 | 10313 | + | 45898 | 47405 | 1507 | Rhamnogalacturonase | x | | | | | 6 | IPR012334,IPR000743 |
| 65142 | 36622 | - | 47630 | 49314 | 1684 | Aldehyde dehydrogenase | x | | | | | 0 | IPR012303,IPR002086 |
| 64971 | 46586 | - | 49852 | 51907 | 2055 | Aromatic and unpolar amino acid permease | x | x | | | | 0 | IPR002293,IPR004841 |
| 71823 | 36620 | - | 52993 | 55245 | 2252 | Cys6-transcription factor | | x | x | | | 0 | IPR007219 |
| 65067 | 30206 | + | 56788 | 58016 | 1228 | Aldo-keto reductase | | | | x | | 0 | IPR001395 |
| 65097 | 10234 | + | 59125 | 60081 | 956 | Alcohol dehydrogenase | x | x | x | | | 0 | IPR002085,IPR013154,IPR013149 |
| 79725 | 10314 | - | 60755 | 61994 | 1239 | Cys6-transcription factor | x | x | x | | | 2 | IPR001138,IPR007219 |
| 65041 | 36617 | - | 62411 | 63580 | 1169 | N2, N2-dimethylguanosine tRNA methyl transferase | | x | x | x | | 0 | IPR000051,IPR013217 |
| 64956 | 36616 | - | 64486 | 65668 | 1182 | Aldehyde dehydrogenase | x | x | | x | | 0 | IPR001395 |
| 109219 | 36615 | + | 66149 | 68189 | 2040 | Hypothetical protein, poorly conserved | | | | | | 0 | |
| 65036 | 30202 | - | 68498 | 70244 | 1746 | Cytochrome P450-dependent alkane hydroxylase | x | x | x | x | | 0 | IPR001128 |
| 109221 | 36613 | + | 70937 | 71776 | 839 | Unknown protein, poorly conserved | | | | | | 0 | |
| 25224 | 36612 | - | 71889 | 74905 | 3016 | Acid trehalase GH65 | x | | | | | 0 | IPR000421,IPR012343,IPR005196,IPR005195,IPR000421 |
| 65172 | 36611 | + | 76106 | 84410 | 8304 | Polyketide synthase class 1, reducing | x | x | x | x | | 0 | IPR006163,IPR001601,IPR000051,IPR000794,IPR009081, IPR000794,IPR001227,IPR013217,IPR013154,IPR013149,IPR002198 |
| 79726 | 36610 | - | 84689 | 85537 | 848 | Hypothetical protein, well conserved | | | | | | 2 | |
